# Mathematical Model of the Layer-by-Layer FFF/FGF Polymer Extrusion Process for Use in the Algorithm of Numerical Implementation of Real-Time Thermal Cycle Control

**DOI:** 10.3390/polym15234518

**Published:** 2023-11-24

**Authors:** Alexander A. Oskolkov, Igor I. Bezukladnikov, Dmitriy N. Trushnikov

**Affiliations:** 1 Department of Welding Production, Metrology and Technology of Material, Perm National Research Polytechnic University, 29 Komsomolsky Prospect, 614990 Perm, Russia; trdimitr@yandex.ru; 2 Department of Automation and Telemechanics, Perm National Research Polytechnic University, 29 Komsomolsky Prospect, 614990 Perm, Russia; corrector@at.pstu.ru

**Keywords:** FFF, 3D printing, additive manufacturing, polymer extrusion, extrusion temperature, thermocycle control, real-time control, automatic control system, mathematical model, simulated model

## Abstract

An approach for improving and maintaining a consistent weld quality of the deposited material during the FFF printing process is proposed. The approach is based on the analysis of the printing process thermal cycle and the real-time nozzle temperature control. The mathematical model of the FFF printing process has been developed with the use of real-time control in the algorithm of numerical implementation. The successful solution of the thermal conductivity problem made it possible to determine segment-wise heating settings for use during the printing process, resulting in a high and stable quality of welding. Comparison of the results of modeling with other well-known mathematical models of the FFF printing process and experimental results showed the adequacy of the proposed model. A maximum deviation of 17.7% between the simulation results and the thermography data was observed. The proposed model was verified using rectangular 3D polylactide shapes printed with and without regulation of the power of the heat source according to the previously estimated settings. The overall quality of regulation, stability of the system, and the PI coefficients of the controller were evaluated using a simulated model of the control system. The results of the experiment fully correspond with the modeling results.

## 1. Introduction

Currently, additive manufacturing technologies are used in various high-tech industries. Additive methods for the production of prostheses, implants and other products of complex geometric shapes made of polymer materials are being actively studied and introduced [1,2,3,4]. A large number of studies are associated with the use of polylactide (PLA) [5,6,7,8], polyether ether ketone (PEEK), polyamides and other biocompatible materials when printing using fused filament/granular fabrication (FFF/FGF) [9,10,11]. 

### 1.1. The Main Disadvantages of FFF 3D Printing Technology

The main disadvantage of FFF technology is the low mechanical properties of products [3,4,12,13,14,15,16,17,18], which limits its use for printing functional products. At the same time, it is possible to have different orientations of the product on the printing platform [12,17,19,20], different strategies for filling the layer [17,18,21], and different directions or angles of laying the thermoplastic beads inside the layer [17,19,20,21,22,23]. If the quality of the welds is low and unstable, this leads to an increase in the anisotropy of the mechanical properties of the product. Inside the layer, this is caused by the difference between the bonding strength of the product along the beads of the material and across it where the welding takes place. The strength of the interlayer weld is less than the strength of the weld between the thermoplastic beads inside the layer and is considered as the main problem [12,14,15,16,24].

Ensuring a high and stable quality of welds can reduce anisotropy and increase the mechanical properties of products [19].

### 1.2. The Mechanism of Material Formation by Welding of Thermoplastic Beads during the FFF Printing Process

In the FFF printing process, the polymer melt (filler material) is applied to the previous layer being in a highly elastic state, or in a glass transition state. At the same time, heating of the previous layer leads to welding of the beads. During the welding process, a short-term partial melting of the surface of the previous layer may occur [3,12,24,25].During the printing process, the extruded bead is pressed against the previous layer by the nozzle and is deformed, exerting pressure on the adjoining bead, and contributing by that to the formation of a weld [25,26,27,28,29]. 

**Figure 1:**
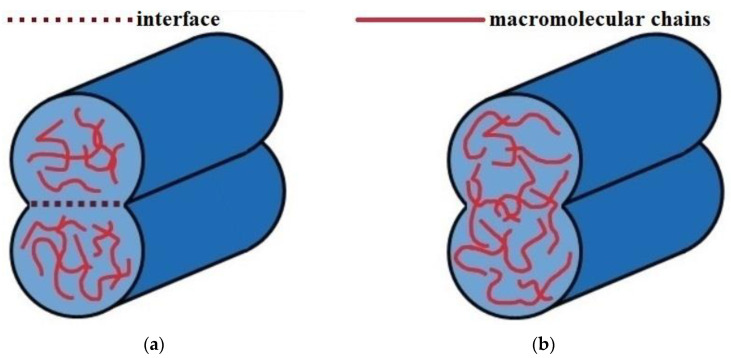
Schematic representation of thermoplastic beads welding in the FFF printing process: (**a**) the process of coalescence (neck growth); (**b**) the process of intermolecular diffusion (healing).

The process of welding of thermoplastic beads is a series of steps, including physical contact and activation of the welded surfaces, the growth of the neck between the thermoplastic beads (coalescence), and intermolecular diffusion (healing) across the interface (contact zone) [30]. In the study of the weld quality, insufficient degrees of coalescence and healing are considered as the main reasons for the low mechanical properties of printed products [3,15,31]. Figure 1 shows a schematic representation of these processes.

Currently, the two-dimensional Frenkel model [32] and its modifications (Eshelby [33], Pokluda [34]) are used to determine the degree of coalescence of thermoplastic beads, describing the coalescence of two spheres of a Newtonian viscous incompressible fluid under isothermal conditions, uniform deformation and absence of external forces (gravity, pressure). The Frenkel model was further developed into the two-dimensional model of the fusion of two infinite cylinders. It was first proposed by Hirao and Tomozawa [35], and fully formulated by Defauchi [36]. The Hopper [37,38] and Balani [39] models are also known, and they describe the coalescence process of two cylinders by solving the Navier–Stokes equations. Under non-isothermal conditions, which are typical for FFF printing, it is necessary to take into account the temperature dependence of the viscosity of the material and the surface tension forces driving the coalescence process, as well as the evolution of the temperature field at the interface of the beads (contact zone) as well [31].

To determine the degree of healing (diffusion), the reptation theory of De Gennes [40] and its modifications (Wool et al. [41]) are used. They describe the mobility of macromolecules during the formation of a bond at the interface of thermoplastic beads. The time required for complete healing is determined by the maximum relaxation time (reptation time) of the thermoplastic material and depends on the welding temperature *T_w_* in the contact zone and its evolution [31]. 

The adequacy of these models was shown in the studies [4,15,42,43,44,45,46,47], the results of which demonstrated the dependence of the mechanical properties of printed products on the degree of coalescence and healing between the thermoplastic beads. These works [31,48,49,50,51,52,53] show modifications of coalescence and healing models to account for the non-isothermal characteristics of the FFF printing process, and demonstrate a more accurate correspondence to the results of the experiments. The following works [29,51,52] describe two-dimensional models of the coalescence of beads in the form of an ellipse, rather than a cylinder, thus considering their deformation when applied to the previous layer.

The processes of coalescence and intermolecular diffusion (healing) between the thermoplastic beads during the FFF printing process occur at a welding temperature *T_w_* in the contact zone above the glass transition temperature *T_g_* with amorphous polymers [19,54,55] or above the crystallization temperature *T_c_* with semi-crystalline polymers [12,19,29,31,54,55]. Moreover, the coalescence process is most intensive at a temperature *T_w_* above the flow temperature with amorphous polymers [42,45,55] and the melting temperature with semi-crystalline polymers accordingly [44,55]. Welding duration and temperature *T_w_* depend on the combination of the temperature of the extrudate *T_ext_* and the previous layer, as well as the conditions of heat removal. At the same time, printing the product layer-by-layer involves cyclic heating of the previous layers, and that affects the quality of the welds between them during the entire printing process of the product.

Thus, information about the thermal cycle of the FFF printing process and its control are critical for improving the mechanical properties of printed products. The influence of temperature *T_w_* and welding duration on the degree of coalescence of the beads of thermoplastic material can be seen from the results of electron microscopy of the cut surfaces of products made with different printing parameters [7,13,14,15,25,27,56,57,58,59,60]. These papers [15,25,57,61] also compare the sizes of the neck between the beads in different areas of the same product, that demonstrates unstable quality of the welds both inside and between the layers. The lower the degree of coalescence, the higher the porosity of the product and the lower its mechanical properties. 

### 1.3. Studying the Thermal Cycle of the FFF Printing Process and Influence of Technological Parameters of Printing

There is a significant number of studies [4,30,62,63,64,65,66,67,68,69,70] devoted to the analysis of the thermal cycle of the FFF printing process and ways to influence it by using the base FFF process parameters, namely the nozzle temperature (extrusion *T_ext_*), temperature of the heated platform *T_pl_*, printing speed (or printing time of a layer), ambient temperature in the printing area. All technological parameters of printing except for the nozzle temperature directly affect the temperature of the previous layer. The analysis of the thermal cycle is usually performed by studying the evolution of temperature values (temperature *T_w_*) at some given point in the contact zone between layers of material using various experimental hardware (thermocouples, pyrometry, thermography) or using mathematical modeling based on primary experimental data. The relation of temperature and time reflects the process of cyclic heating in the contact zone when applying subsequent layers of material. The result of most studies is the shift and maintenance of temperature *T_w_* values, above temperatures *T_g_* or *T_c_* during cyclic heating of the layers of the material to the possible extent. It is necessary to keep welding temperatures *T_w_* as high as possible without polymer degradation. Higher values of *T_w_* increase not only the duration of welding but also the rate of growth of the neck due to the influence of temperature on the viscosity of the material [31,48,49,71]. This also reduces the time required for both the completion of the coalescence process and intermolecular diffusion. At the same time, as the temperature in the contact zone cools down and decreases, the processes of neck growth and intermolecular diffusion slow down. The authors of [31] proposed to use diagrams of the dependence of the degree of coalescence and healing on temperature and time under isothermal conditions to estimate the required duration and temperature *T_w_* of welding on the example of PEKK material. Moreover, the results of the numerical simulation showed that with an increase in the ambient and extrusion temperature *T_ext_*, as well as printing speed of product made of PEKK, it was possible to achieve an increase in the degree of coalescence between the beads of the material from 0.11 to 0.28. At the same time, the process of intermolecular diffusion proceeds and completes much faster than the growth of the neck. 

Another measure is to maximize the duration of the welding process in the FFF printing process [3,4,7,15,16,29,66], that leads to increase in the time at which the temperature *T_w_* in the contact zone above *T_g_* or *T_c_*. The authors of [4] state that the shift of only the peak values of temperature *T_w_* above temperature *T_g_* on the example of ABS and TPU during cyclic heating made it possible to increase the strength of the welds between plates made of these materials. This result was achieved by increasing the temperature of the heated platform *T_pl_*, extrusion temperature *T_ext_* and printing speed. The rise in temperature *T_pl_*, from 303 to 341 K provided an increase in the strength of the welds from 0.86 to 1.66 MPa (93%). The rise in the temperature *T_ext_* from 513 to 533 K provided an increase in the strength of the welds by 15.3%. And the rise in the printing speed from 8 to 12 mm/s provided an increase in the strength of the welds by 5.6%. The authors of [16,66] proposed the concept of equivalent welding time, in which the welding time of a non-isothermal FFF printing process is recalculated for the selected reference temperature *T_ref_* = 503 K in the isothermal case. The authors thus established the dependence between the strength of the weld and welding time during the printing process. An increase in the temperature *T_ext_* from 483 to 543 K and the printing speed from 3 to 100 mm/s, when printing an ABS product, made it possible to increase the equivalent welding time from 0.01 to 0.12 s and the strength of the welds between the layers by 3 fold. Platform temperature *T_pl_* remained constant and equal to 383 K. Thus, the duration of welding correlates with the welding temperature *T_w_*. For a better understanding of the influence of the technological parameters of FFF printing on the mechanical properties of products, let us consider the results of a number of other studies.

In [12], reducing of the printing time of the layer led to a shift and maintenance of the temperature values *T_w_* above the temperature *T_c_* on the example of PLA, which provided an increase in the strength of the bond between the layers by 23%. In [13], an increase in the impact strength of PLA products by 113% is reported in the case of an increase in temperature *T_pl_* from 303 to 433 K. It is known from [19] that when products made of PA1012 were printed, an increase in the temperature *T_ext_* from 473 to 493 K provided an increase in the strength of the bond between the layers from 12 to 26 MPa. The relationship between the base FFF process parameters and the outcoming mechanical characteristics is also studied in [7,25,56]. 

The decrease in the welding temperature *T_w_* as it moves away from the platform, as well as the unevenness of the temperature field in the volume of the product (along all axes) during the printing process is noted in many studies and is presented as the results of thermography or pyrometry [12,57,63,67,68,72], and the results of electron microscopy of products [7,13,14,15,25,27,56,57,58]. This phenomenon is explained by a change in the heat removal conditions during the printing process and cannot be compensated only by a change in the printing speed, temperature *T_pl_* or the environment temperature. The lower layers are exposed to temperature for a longer time, which provides a greater degree of coalescence and healing between the beads of the material [13,15,73]. The effect of platform temperature *T_pl_* on the welding quality is limited by the size of the product, namely its height (Z axis). 

Control of the thermal cycle of the FFF printing process by changing the temperature of the platform *T_pl_* or the printing speed is not universal and loses its effectiveness as the size of the product increases both in the XY plane and along the Z axis. The problem of printing products of complex geometric shapes and large-sized products in constantly changing heat removal conditions is directly set in the studies [3,16,17,31,73,74,75,76,77]. 

The limited possibilities of improving the mechanical properties of products by changing the base FFF process parameters led to the use of additional energy sources, such as ultraviolet lamps or lasers for local heating of the previous layer to reach the glass transition temperature *T_g_* or crystallization temperature *T_c_* before applying the next layer [78,79,80,81]. The presented results demonstrate the high efficiency of the proposed methods; however, the lack of regulation of the power of the sources does not enable considering the change in the conditions of the heat removal during the printing process. This leads to local overheating of the previous layers and additional thermal deformations of the products [12,65,69,79,80,82,83].

### 1.4. Mathematical Modeling of the FFF Printing Process

Currently, mathematical modeling is used both for the study of temperature fields and the analysis of the thermal cycle of the FFF printing process, and for the assessment of residual stresses and thermal deformations of the product.

Study [84] shows a finite element model of the FFF printing process, which uses the technologies of “killing” and “aliving” of the elements. The deposition process is modeled by the initial “killing” of all elements of the voxel model of the product, followed by the “aliving” of that part, where the volumetric heat source moves along the trajectory of the extruder. The paper analyzes the influence of the key parameters of the deposition process on the temperature field, the distribution of stresses and thermal deformations. The optimal parameters of deposition in an environment with a given temperature were determined and the results of the modeling were verified. It is revealed that the greatest influence on the magnitude of thermal deformations is exerted by the temperature in the sealed chamber of the 3D printer. A similar model is described in [85], and it was used to study the temperature field during deposition of product and selection of the deposition speed. A similar method of modeling the 3D printing process was also used for metal deposition [86]. The third similar model is described in [87], and was used to study the distribution and magnitude of residual stresses. The paper proposes two approaches to modeling the process of layer-by-layer deposition. The first approach is about simulating the application of a bead of material by sequentially activating elements with an initial temperature corresponding to the extrusion temperature *T_ext_*, in accordance with the trajectory of the extruder, and the second one is about “aliving” the whole layer of the model at once. The authors conclude that although both approaches enable studying temperature fields with sufficient reliability, with the “aliving” of the whole layer it is not possible to study the distribution of residual stresses. The study in [88] shows that the technology of “killing” and “aliving” elements with a given initial temperature of activated elements is used in the same way as [87] to study the process of laying out one bead of material, using ABS as an example, and [89] shows laying out composite material and evaluating thermal deformations of a printed product. A similar model was used in [90] to study the temperature field and thermal deformations when printing from ABS, and in [91] to study the thermal cycle when printing from PLA, followed by verification of the model by experiment. As a result, the experiment showed that the maximum welding time is achieved at the maximum layer height, temperatures *T_ext_* and *T_pl_*, and low print speed. However, to reduce thermal deformations and stresses, it is recommended to reduce the temperature *T_ext_* and layer height.

Mathematical models [92,93,94,95] of the process of laying out a bead of molten thermoplastics when solving problems of polymer hydrodynamics with the original numerical method proposed in [92] for solving problems of layer-by-layer deposition are of particular interest. Such models enable us to study the process of layer formation as a result of the coalescence of beads and the formation of a multilayer structure in more detail. It is also possible to perform a more accurate assessment of the influence of the technological parameters of the layer-by-layer deposition process on the formation of the bead and the whole product, and to identify the features of deposition with specific types of thermoplastics.

In [96], the process of extrusion of a single bead of material onto a platform is also studied, but without taking into account hydrodynamic effects. The simulation results demonstrate the temperature distribution in the material (PLA), both inside the extruder and after its extrusion onto the platform during the movement of the extruder.

Two-dimensional and three-dimensional mathematical models are described in [31,67,97,98]. Their purpose is to study the thermal cycle of the FFF printing process, as well as the coalescence and intermolecular diffusion of the applied material with the previous layer or bead to determine the printing parameters that ensure the highest quality of the welds.

The approach presented for controlling the process of three-dimensional printing with the regulation of the heating power of the polymer in the nozzle based on the results of numerical simulation uses a specially developed algorithm. Such a method has already proven itself well when there is deposition of metal materials [99]. It is proposed to use the results of numerical studies of the temperature fields of the product (sample) in the printing process using real-time control methods in the algorithm of numerical implementation to determine the parameters of the thermal input required for the layer or segment of the layer. In [100,101], the developed specialized extruders with induction heating are presented. They significantly reduce the inertia of heating and cooling the nozzle. Their use enables adjusting the extrusion temperature *T_ext_* in a predetermined fashion directly in the FFF printing process, taking into account the data of changing heat removal conditions. 

## 2. Materials and Methods

The induction extruder with a lightweight nozzle (<1 g) developed by the team of authors [100] provides the possibility of rapid control of the extrusion temperature *T_ext_* to regulate the thermal cycle of the FFF printing process. It will increase the stability of welding quality and improve the mechanical properties of products and reduce thermal deformations. A more uniform distribution of residual stresses will be achieved. 

**Figure 2:**
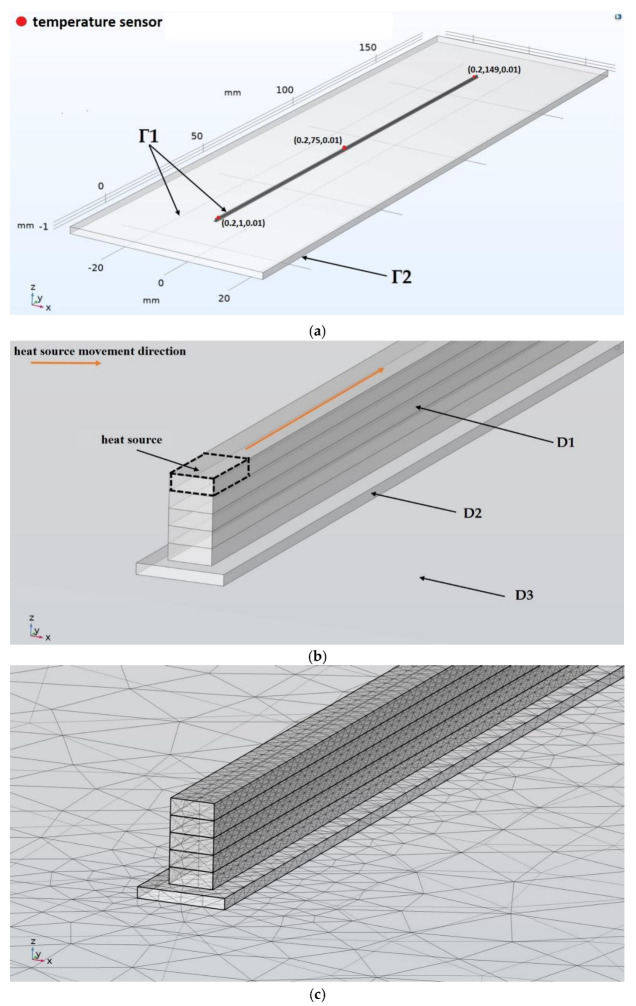
Computational scheme of the sample deposition process: (**a**) boundary conditions and location of the temperature sensors; (**b**) computational domain; (**c**) division of the computational domain into finite elements of a tetrahedral shape.

To implement this approach, a mathematical model of the FFF printing process (layer-by-layer deposition) was developed using real-time control of the heat source power. The volume of the polymer melt pool was used as a controlled parameter in the feedback loop. The computational Scheme is shown in Figure 2a.

The proposed model was verified using rectangular 3D polylactide shapes (samples) printed with and without regulation of the power of the heat source according to the previously estimated settings. The experimental sample is a wall with a width of one bead and other dimensions specified.

The deposition of the samples was performed using an extruder of our own design, the device and the principles of operation of which are described in [100], installed on an FFF 3D printer of standard design. A filament/wire made of PLA with a diameter of 1.75 mm manufactured by SEM3D was used for deposition [102]. 

During experiments, before starting the printing process, the platform and the extruder are warmed up to the set temperatures *T_pl_* = 313 K and *T_ext_*. At the beginning of the printing process, a polymer substrate is formed (domain D2 in Figure 2). It is applied to the glass platform, forming a layer of polymer material of the required size smoothed by the nozzle, tightly adjacent to the platform. All of the following together with the use of an adhesive solution (polyvinylpyrrolidone (PVP) based glue), ensures the fixation of the manufactured sample on the platform. Heating the platform to the temperature *T_pl_* ensures that the polymer material is maintained in a highly elastic state after it is squeezed out of the extruder and, thus, the best adhesion quality with minimal influence of the platform temperature on the sample layers. To exclude the influence of the extrusion quality variation and extrusion stability on the formation of samples, the process of every bead/layer extrusion begins shortly (approx. 0.7 s) before the following movement of the extruder and ends shortly after. The effect of that is observed in the form of blobs/pillars on the edges of the samples (walls). All samples were printed with a nozzle with a forming hole diameter of 0.3 mm, which gave a sample (wall) width of approximately 0.4 mm. The layer height of all samples is 0.15 mm.

The non-stationary thermal conductivity problem in a rectangular sample was solved applied to the process of layer-by-layer deposition of a semi-crystalline polymer PLA. The numerical implementation of the mathematical model was performed in the applied software package Comsol Multiphysics (https://www.comsol.jp/, accessed on 20 October 2023). 

In this work, a numerical study of the temperature field was performed for the following cases: a single bead, a single bead on a platform with a polymer substrate, a multilayer structure (sample/wall) with a width of one bead of various lengths on a platform with a polymer substrate, and a multilayer structure (sample/wall) with a width of two beads on a platform with a polymer substrate. The presented description of the mathematical model is valid for all cases, except for the first one, where there is no platform with a polymer substrate and associated boundary conditions. Variation in some printing parameters is also typical for all cases.

When creating the mathematical model, the following assumptions were made:

- Neglecting phase transitions;
- Neglecting hydrodynamic effects;
- Neglecting viscous dissipation;
- Neglecting deformations.

The computational domain is represented in Figure 2 by the finite volume of the sample (D1), the polymer substrate (D2) and the platform (D3). At the start, we have performed modeling with different sizes of mesh, but to optimize the performance and reduce calculation time, the final mesh size was selected with the criteria that further reducing of the mesh will not lead to a significant reduction in error. The layers (domain D1) were divided into finite elements of a tetrahedral shape with a maximum element size of 0.15 mm (1.8 mm for domains D2 and D3).

The deposition of a sample with a length of 150 mm and a height of 0.75 mm (5 layers) on a polymer substrate with a size of 0.8 × 150.4 × 0.1 mm^3^, which is already deposited on a heated glass platform with a size of 210 × 60 × 2 mm^3^ (depending on the size of the sample, the dimensions of the polymer substrate were +0.2 mm on each side of the sample, and the dimensions of the platform +30 mm), was modeled. The layer height was *l_z_* = 0.15 mm, and the printing speed is equal to the speed of idle movements $v_{y}$. The layers were printed in one direction along the *y* axis. The presence of a heat source with a height of *l_z_* was set using a movable indicator function (1):

(1) $$I\left(x,y,z,t\right)=\left(h\left(z+l_{z}\right)-h\left(z\right)\right)\left(h\left(y-v_{y}t\right)-h\left(y-v_{y}t-l_{y}\right)\right)\left(h\left(x\right)-h\left(x-l_{x}\right)\right)$$

where $h\left(s\right)=\{0,s<0,s\geq 0\}$ is the Heaviside function, and *l_y_* = *l_x_* = 0.4 mm are dimensions of the heat source approximated by a parallelepiped, which correspond to the width of the sample in the computational scheme and the width of the sample obtained experimentally by deposition with a nozzle with a hole diameter of 0.3 mm. In the computational domain, the equation of thermal conductivity [103] (2) was solved:

(2) $$\rho c_{p}\frac{\partial T}{\partial t}=\nabla \cdot \left(\lambda \nabla T\right)+q,$$

where T is the absolute temperature, ρ is the material density, $c_{p}$ and λ are specific heat capacity and thermal conductivity, ∇ is the Hamilton operator, and q is the specific power of the volumetric heat source. At the initial time of the deposition process, all layers of the sample are “dead”. “Killing” is implemented through the degradation of the thermophysical constants of the material: λ(a)→0. For the sample material: λ = λ(a). The “aliving” of the layer occurs when the heat source is moved to this layer: $a\left(z\right)=\left(h\left(z+l_{z}\right)-h\left(z\right)\right)$ (3).

(3) $$\lambda \left(a\right)=\left\{\begin{array}{c} 10^{-8},\;a\leq 0,\\ \lambda_{p},a>0. \end{array}\right.$$

When “aliving” the layer: $\lambda \left(a\right)=\lambda_{p}$, where $\lambda_{p}$ is the specific thermal conductivity of PLA. The conditions of convective and radiative heat transfer with the environment were set on the boundary Г1 of the computational domain: $-\lambda n\cdot \nabla T=k\left(T-T_{0}\right)+\varepsilon_{T}\sigma_{CБ}\left(T^{4}-T_{0}^{4}\right)$. Here: $T_{0}$ is the absolute ambient temperature; *k* is the coefficient of heat transfer from the surface to the environment; σ_SB_ is the Stefan–Boltzmann constant; ε_t_ is the coefficient of thermal radiation; **n** is the normal vector on the boundary. The conditions are set ${\left.T\right|}_{Г2}=313\;K$. Initial conditions were set for domains (D1), (D2) and (D3) ${\left.T\right|}_{t=0}=T_{pl}=313\;K.$ $T_{pl}$ is the initial temperature of the platform and the polymer substrate. Based on the results of [67], it was decided to consider the thermophysical properties of PLA as constant, since their dependence on temperature has a negligible effect on the results of numerical simulation; the authors [67] also mention the need to take into account radiative heat transfer in the absence of forced convection. The thermophysical constants of PLA accepted in the model are shown in Table 1. The thermophysical constants of glass are given in Table 2.

Internal surfaces of the interface, melting and crystallization fronts were not considered in the model. The problem of thermal conductivity was solved using an implicit integration scheme with a time step τ=0.01 s.

Volumetric heat source in the form of a rectangular parallelepiped in size $l_{x}\times l_{y}\times l_{z}$ and volume $V_{*}$ moved at a speed of $v_{y}$ along the axis y. Power of the heat source in volume $V_{*}$ calculated according to the Formula (4):

(4) $$P(t)=\int_{-\infty }^{\infty }\int_{-\infty }^{\infty }\int_{-\infty }^{\infty }q(x,y,z,t)dxdydz=V_{*}q_{*}(t),$$

where $q\left(x,y,z,t\right)=I\left(x,y,z,t\right)q_{*}\left(t\right)$. It was believed that the specific power of the heat source: $q_{*}\left(t\right)=P\left(t\right)/V_{*}$ evenly distributed in volume $V_{*}$ at the given moment of time. To implement the algorithm, a macro was written in the Comsol Multiphysics package that ensures that the heat source follows a given trajectory.

At the last stage of the modeling, the power of the heat source was automatically adjusted for the uniform heating during the deposition process, similar to the study in [99]. Feedback for power control was implemented by measuring the volume of the melt pool.

To weld the beads of semi-crystalline thermoplastics, i.e., to grow the neck, the welding temperature *T_w_* is required above the crystallization temperature *T_c_*, which for PLA is approximately 378 K according to various sources. The glass transition temperature *T_g_* is approximately 333 K, and the destruction temperature is approximately 533 K. At the same time, the temperatures of the melting interval of PLA are known, and the temperature of complete melting of the crystalline phase *T_l_* is approximately 438 K, and the temperature of the beginning of melting or partial melting *T_s_* is approximately 418 K. The given temperature values are taken from the following sources [7,12,13,14,27,44,65,68,73,106].

The volume of the melt pool *V_s_* was determined by the partial melting temperature of PLA: $T\left(V_{s}\right)\geq T_{s}$. The peculiarity of polymers in comparison with metals is low thermal conductivity and low melting point in the case of PLA. As a result of which the melt pool is poorly localized, i.e., significantly distributed inside the layer, which complicates the control of its volume. To solve this problem, an additional condition was introduced that restricts the computational domain of the actual volume of the pool not only by melting temperature $T_{s}$ but also in the area around the heat source moving along the *y* axis. The following movable indicator function was used for it: $b\left(y,t\right)=\left(h\left(y-v_{y}t+1\right)-h\left(y-v_{y}t-l_{y}-1\right)\right)$. The control of the deposition process at each time step was performed according to the discrepancy: $u\left(t\right)=\frac{\left(V_{s}\left(t\right)-V_{**}\right)}{V_{**}}$ using the proportional-integral (PI) control laws (5):

(5) $$P\left(t+\tau \right)=P\left(t\right)-K_{p}P\left(t\right)u\left(t\right)-K_{I}P\left(t\right)\int_{0}^{t}u\left(t\right)dt,\cdots \cdots P\left(0\right)=P_{0}$$

At the same time, $V_{**}$—the set value of the melt pool volume—was determined from solving the problem of thermal conductivity by applying a bead of a material of a given temperature to a platform with a polymer substrate without using the PI controller. $K_{p}$ and $K_{I}$ are the coefficients of the PI controller. From [3,59,107,108,109], we know that the minimum extrusion temperature of PLA material, at which the welding of the layers is provided, is 453 K, and the optimal temperature is 483–493 K. Moreover, the destruction temperature of PLA is approximately 523–553 K. The desired volume of the melt pool $V_{**}$; therefore, we will determine for the case of applying a thermoplastic bead with a temperature *T_ext_* = 493 K.

## 3. Results and Discussion

### 3.1. Determination of the Power of the Heat Source That Provides Heating of the Material Bead to the Required Extrusion Temperature

**Figure 3:**
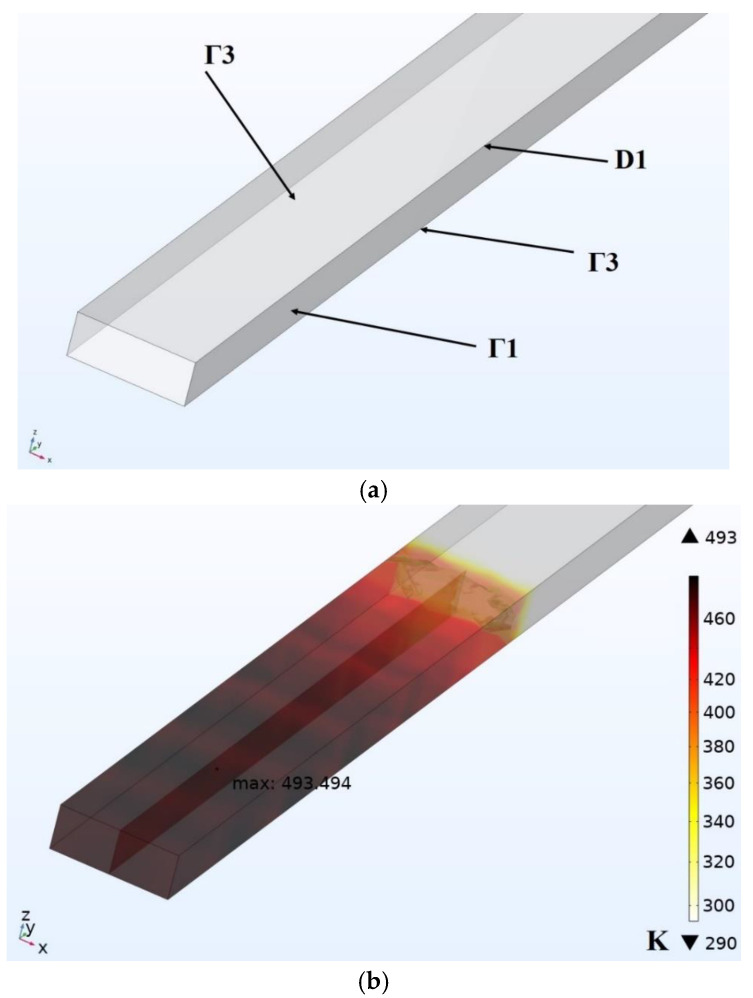
Modeling of the deposition process of a single bead: (**a**) computational scheme; (**b**) the result of solving the thermal problem at the moment t = 0.1 s.

The process of applying a new material of a given temperature *T_ext_* is modeled by a volumetric heat source. To simulate the process of applying a new layer of thermoplastic material, first of all it is necessary to determine the power of the heat source $P_{0}$, which is required to ensure the required temperature of a single bead while moving the heat source at a given speed corresponding to the linear printing speed. In a real extruder, the thermoplastics are heated to a set temperature *T_ext_* under conditions of no heat transfer between the thermoplastics and the environment (air). In the proposed model, the bead is heated to the required temperature not in advance in the extruder, but already in contact with the platform or the previous layer of material, i.e., after extrusion, which means that convective and radiative heat transfer with the environment (air) must be taken into account when determining the power of the heat source. It is known from [110] that after the bead is extruded in air, its temperature at a distance of 0.4 mm from the nozzle decreases by less than 1%. Therefore, in this subtask, it is legitimate to heat the bead directly to the extrusion temperature *T_ext_*. When solving this subtask, it is also necessary to exclude heat transfer with the platform on which the deposition is performed. Therefore, thermal insulation conditions (Г3) are set at the lower boundary of the bead. The latter is necessary for the reason that under conditions of contact and heat transfer with the platform, the temperature distribution in the material bead during heating will be uneven. Therefore, it will become difficult to determine the temperature to which it is necessary to heat the bead. Since at this stage of modeling we are only interested in the temperature in the area occupied by the heat source, the thermal insulation conditions (Г3) are also accepted at the upper boundary of the bead, because it is assumed that in the place occupied by the heat source, the extrusion of the material heated to a given temperature *T_ext_* is performed, and with further modeling of multilayer deposition, the upper boundary of the bead will be covered with a new layer of thermoplastics. The maximum temperature in the area through which the heat source has passed is measured. The computational scheme of this modeling stage for the extrusion temperature *T_ext_* = 493 K, $v_{y}$ = 40 mm/s with the previously specified dimensions of the bead is shown in Figure 3.

As a result of solving the thermal problem, it was found that to ensure *T_ext_* = 493 K at $v_{y}$ = 40 mm/s for a bead of the specified size, the power of the heat source is required $P_{0}$ = 1.05 W. A similar task was solved for a number of other combinations of deposition parameters, which will be required further. The results are shown in Table 3.

### 3.2. Modeling of the FFF Printing Process of a Long Sample according to the Computational Scheme 2 for T_ext_ = 493 K

By determining the power value of the heat source $P_{0}$, which is necessary to ensure the required temperature *T_ext_* = 493 K at $v_{y}$ = 40 mm/s, we should conduct a numerical study of the temperature field during the deposition of the sample according to the computational scheme shown in Figure 2 in an open-loop control system, i.e., without the use of a PI controller.

**Figure 4:**
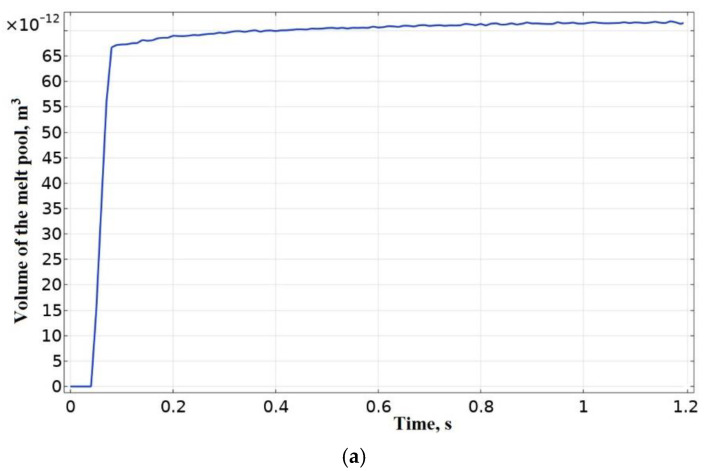

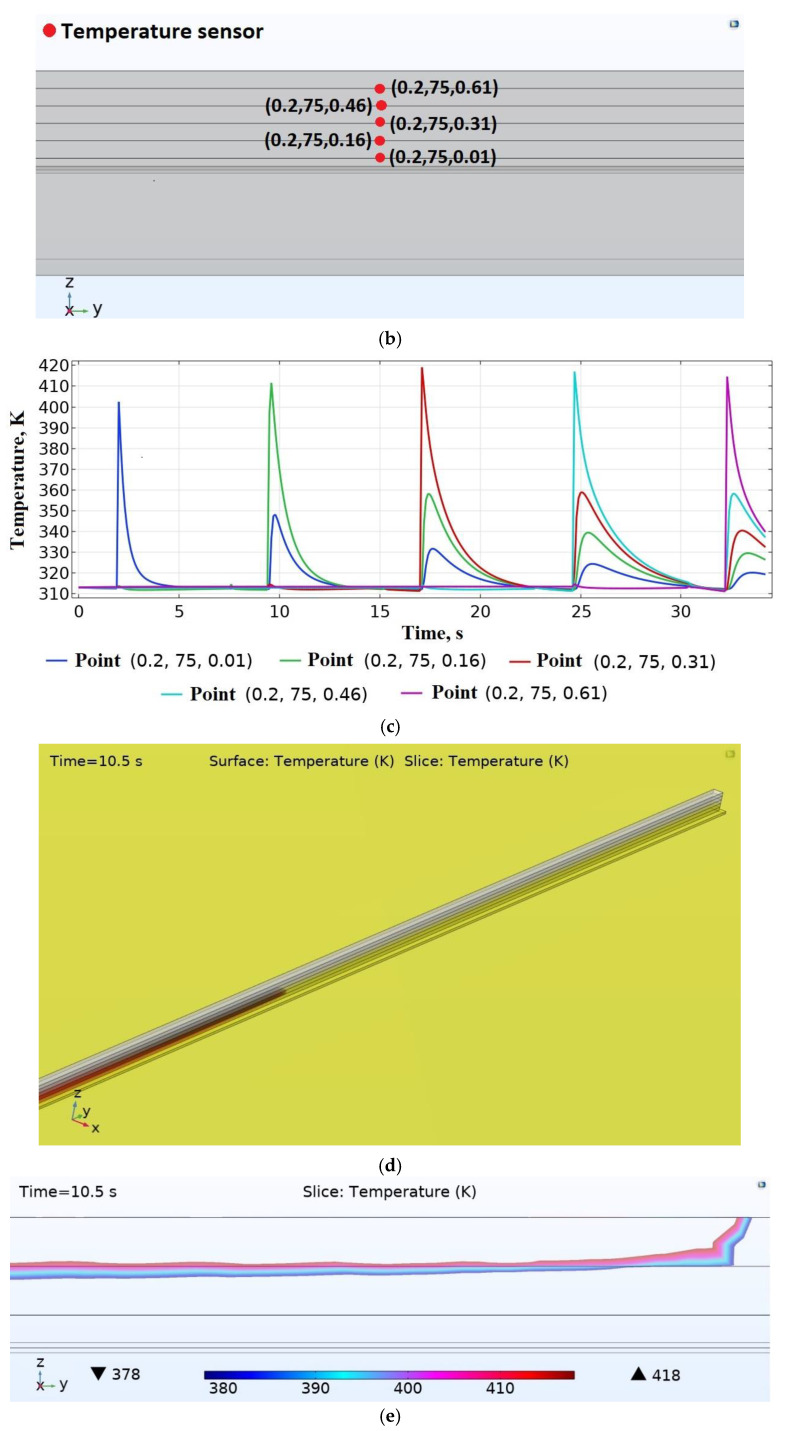
The result of solving the thermal problem: (**a**) the estimated volume of the melt pool; (**b**) location of temperature sensors; (**c**) the evolution of temperature values at given points; (**d**) the thermal field at moment t = 10.5 s; (**e**) isotherms of temperatures *T_s_* and *T_c_*.

The solution of the thermal problem made it possible to determine the volume of the melt pool $V_{**}$ = 7.1 × 10^−11^ m^3^ when there is deposition of the first layer of thermoplastics on a platform with a polymer substrate, as shown in Figure 4a. In addition, welding temperatures *T_w_* were determined between the first layer and the platform with a polymer substrate, the first and second layers. The latter made it possible to compare the data obtained with the results of numerical simulation of the process of laying out a bead of molten thermoplastics when solving a hydrodynamic problem [92] and to obtain a primary assessment of the adequacy of modeling the FFF printing process using a volumetric heat source. A graph of the evolution of temperature values for points in the middle of the sample length (75, 0.2, 0.01:0.16:0.31:0.46:0.61) is shown in Figure 4b,c. It demonstrates the change in the initial welding temperature *T_w_*, as well as the change in the temperature of cyclic heating (evolution *T_w_*) for different layers, related with a change in conditions of heat removal as it is moving away from the platform and increasing the surface area and sample mass. Figure 4d shows the result of a numerical study of the thermal field at the moment t = 10.5 s, and Figure 4e visualizes the isotherms of temperatures *T_s_* and *T_c_* at the moment t = 10.5 s, enabling estimation of the distribution of the melt pool in the volume of the bead and the melting depth of the previous layer. It can be seen that melting of the surface of the previous layer does not occur; however, the temperature is *T_w_* > *T_c_*, which ensures welding of the layers.

Currently, the numerical solution takes approximately 20-fold longer than printing a full-size sample, which is generally unacceptable. However, at the present stage of this study, this problem does not matter, since the main point for this stage was to investigate the patterns of occurrence of defects caused by overheating and insufficient heating of the material and to find a way to avoid such defects. In the future, various methods can be used to reduce computational costs, for example, using voxel-based FEM modeling with the technologies of “killing” and “aliving” of the elements, improving computational algorithms using neural networks, AI, etc.

### 3.3. Comparison of the Results of Modeling the FFF Printing Process Using a Volumetric Heat Source with Other Well-Known Mathematical Models of the FFF Printing Process 

According to Figure 4c, the welding temperature *T_w_* in the contact zone of the first and second layers was approximately 413 K. The printing time of the layer in this case is 3.8 s. In [92], when applying 0.7 mm high bead to each other with a layer printing time of 3 s, *T_ext_* = 488 K and $v_{y}$ = 10 mm/s, as well as ignoring radiation heat transfer and small differences in the accepted values of thermophysical constants for PLA, the temperature *T_w_* between the first and second layer was 423–443 K. The difference in the modeling results can be explained by a much lower degree of cooling of the previous layer in [92].

**Figure 5:**
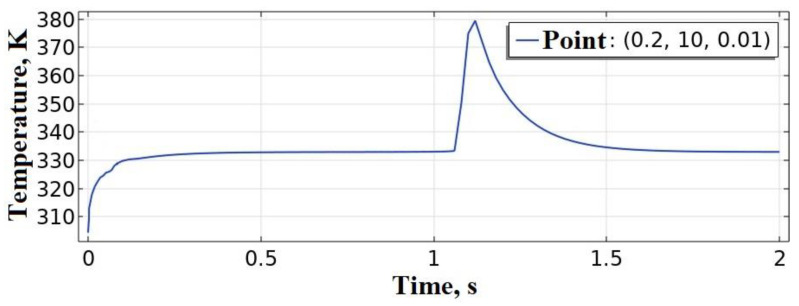
Evolution of temperature values at a given point.

A mathematical model of the extrusion process of a single bead onto a platform is described in [96], but without solving the hydrodynamic problem. Deposition was performed with PLA material at *T_ext_* = 493 K, $v_{y}$ = 10 mm/s, *T_pl_* = 333 K, while the platform is made of steel. The thermophysical constants accepted in the model for PLA differ slightly from those adopted in our model, and for steel: $c_{p}=440$ J·kg^−1^·K^−1^;$\lambda_{p}=76$ W·m^−1^·K^−1^. *k* = 40 W·m^−2^·K^−1^ at the boundaries of the extruder. Radiation heat transfer is ignored. The authors [96] found that the temperature in the contact zone of the bead with the platform was approximately 393 K. The technological parameters of printing from [96] were transferred to the proposed model, the computational scheme of which is shown in Figure 2 with the following changes: only the first layer was kept, the thickness of the polymer substrate was reduced to 0.01 mm, the platform material was replaced with steel from [96], the power of the heat source according to Table 3 was $P_{0}$ = 0.27 W. As a result of solving the thermal problem, the temperature was measured at the point (0.2, 10, 0.01), which was 380 K (Figure 5), which is 12% less than in [96]. This deviation can be partially explained by the absence of convective and radiation heat transfer conditions at the bead boundary in [96].

### 3.4. Verification of the Proposed Mathematical Model Using Previously Known Experimental Results and Thermography Data

The paper in [27] provides a detailed description of the experiment on deposition a rectangular sample (wall) with a width of two PLA beads and the results of thermography, which determined the welding temperature on the outer surface of the contact zone between the last and penultimate layer. This makes possible to verify the proposed mathematical model using experimental data of [27] and to reliably assess its adequacy from the quantitative side. In [27], the height of the layer *l_z_* was chosen equal to 0.3 mm, the width of the bead *l_x_* equal to 0.5 mm (the width of the layer equal to 1 mm), *T_ext_* = 463 K, $v_{y}$ = 10 mm/s, and *T_pl_* = 303 K. The wall height was 3.9 mm or 13 layers, and its length was 40 mm. Based on the analysis of the data given in [27], it can be unambiguously concluded that the deposition was performed in one direction alternating the left and right bead, and the speed of idle movements was 15 mm/s. The welding temperature *T_w_* at the measuring point was approximately 393 K.

**Figure 6:**
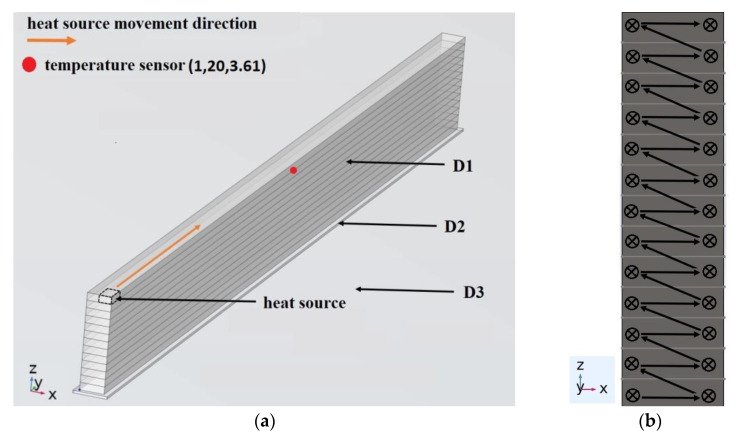

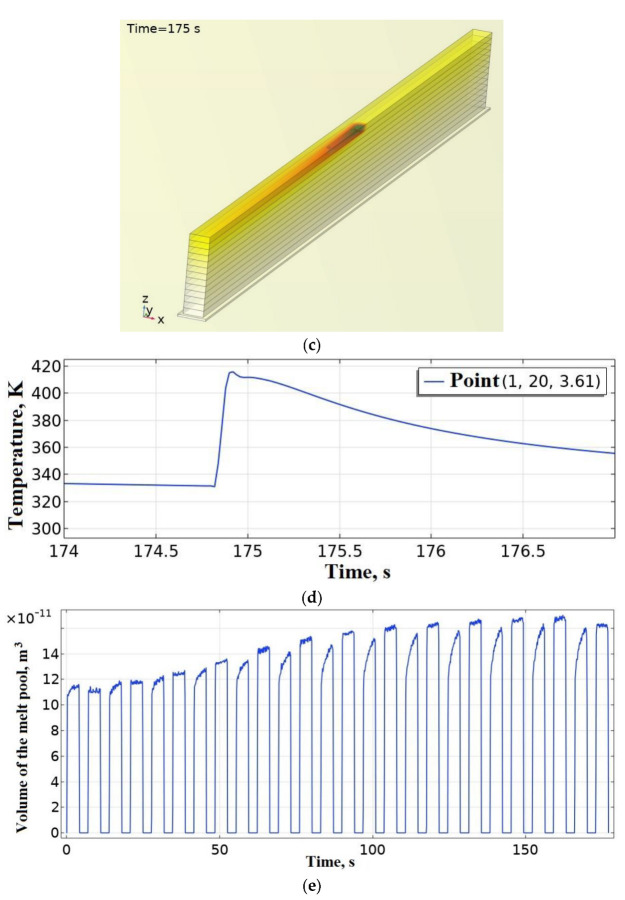
Modeling of double wall deposition: (**a**) computational scheme; (**b**) the order of deposition of material beads; (**c**) thermal field at time t = 175 s; (**d**) evolution of temperature values at point (1, 20, 3.61); (**e**) evolution of the volume of the melt pool.

The computational scheme of the model is shown in Figure 6a. The boundary conditions are similar to the first model (Figure 2) except for the temperature of the platform and the polymer substrate. The conditions are set ${\left.T\right|}_{Г2}=303\;K$. Initial conditions were set for domains (D1), (D2) and (D3)${\left.T\right|}_{t=0}=T_{pl}=303\;K.$ The power of the heat source according to Table 3 is assumed to be equal $P_{0}$ = 0.56 W. Figure 6b shows the order of deposition of material beads. All beads were deposited in one direction. On each layer, the left bead was deposited first.

As a result of solving the thermal problem, a graph of the evolution of temperature values at the point (1, 20, 3.61) was obtained. It demonstrates the temperature *T_w_* = 418 K, which is 17.7% higher than the temperature obtained by the results of thermography in [27]. The main reason for the deviation in this case is the activation of the entire deposition layer at once. Each layer is deposited in two beads but there is no boundary between beads inside layer. So, there are no corresponding boundary conditions (convective and radiation heat transfer with the environment) in the interface between them. The conditions of heat transfer with the environment are set only at the boundaries of the layer, and not for a separate bead. Moreover, the conditions of heat transfer with the environment are set only for the upper boundary of the last layer but not between layers. For these reasons, the modeled cooling rate of the sample is lower than it should be. In this example, the temperature between the last two layers is measured, so there could be an additive error, that grows over time and with each new layer as the excess heat accumulates in the system.

The results of verification using experimental data of [27], as well as the comparison with the results of numerical simulation of [92,96], enable us to conclude that the proposed model is adequate both qualitative and quantitative.

Figure 6e shows a graph of the evolution of the volume of the melt pool. It can be seen that the system reaches the steady state by approximately the 10th layer, which is due to a change in the conditions of the heat removal. In addition, the volume of the pool at the beginning of the layer is always lower than at the end of the layer, which indicates an uneven cooling of the wall and a gradual accumulation of heat at the end of the wall, which is caused by a change in the mass of the wall, i.e., the cooling mass at the beginning of the layer is less than at the end, which affects the cooling rate and temperature *T_w_*. The above result indicates the need to regulate the power of the heat source to ensure stable quality of the welds, reduce the degree of overheating and insufficient heating in the contact zone and the previous layers on the whole.

### 3.5. Modeling of the FFF Printing Process of a Short Sample 

To study the thermal effects associated with overheating and insufficient heating, an additional stage of modeling was performed in order to further verify the results based on our own experiment on deposition samples with a width of one bead. When modeling the printing process, we consider two cases. Modeling of the printing process of short and long samples. We are doing so due to the fact that the effect of material overheating is more pronounced when printing small products, i.e., short samples. At the same time, the effect of insufficient heating is more pronounced when printing larger products, i.e., long samples. Thus, considering the process of printing short and long samples, we study the effects of overheating and insufficient heating of the material and compare calculated and experimental data.

**Figure 7:**
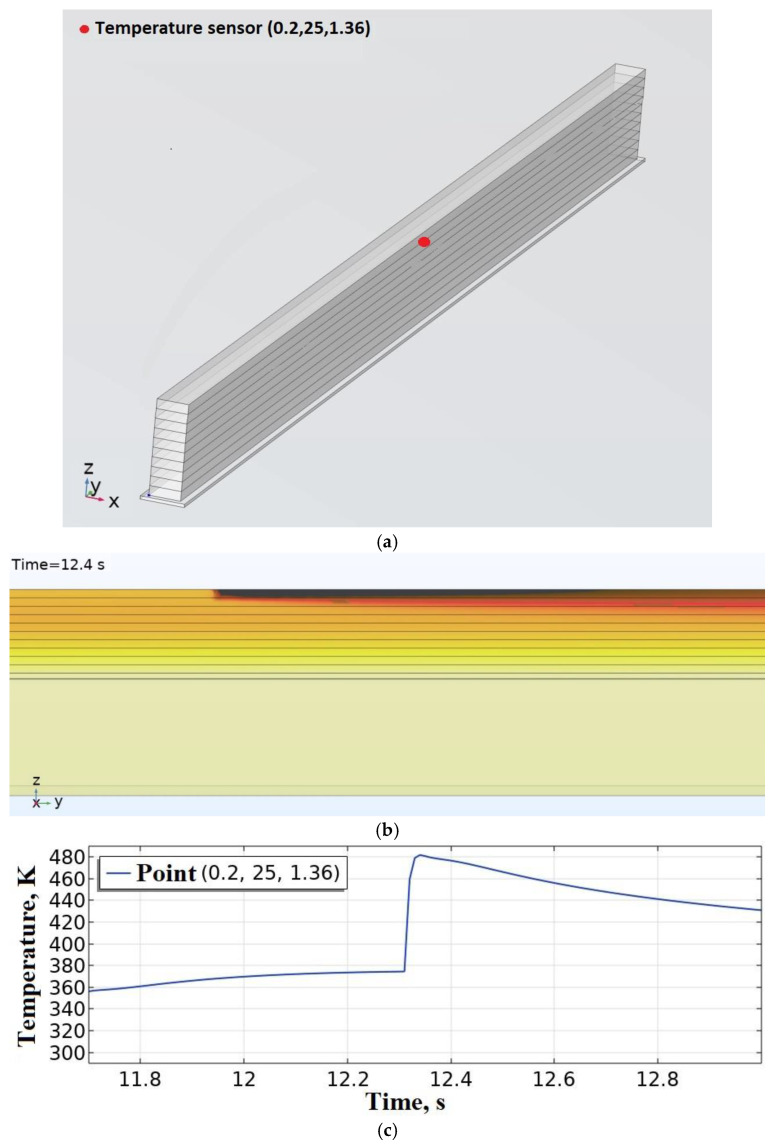

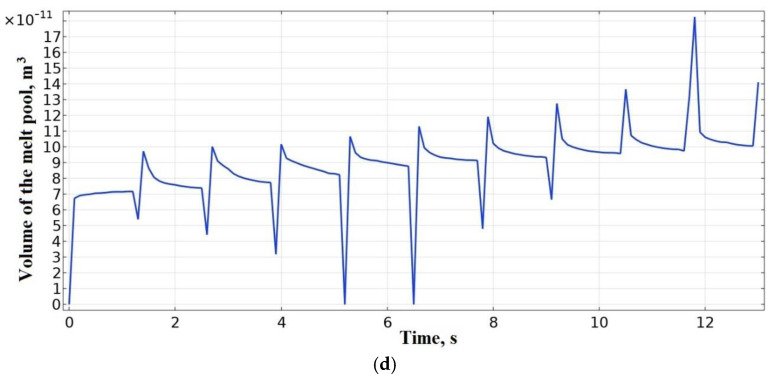
Modeling of short sample deposition: (**a**) location of temperature sensor; (**b**) thermal field at time t = 12.4 s; (**c**) evolution of temperature values at the point (0.2, 25, 1.36); (**d**) evolution of the volume of the melt pool.

The deposition of the short samples was modeled. They were made of PLA with a height of 1.5 mm (10 layers) and a width of one bead 0.4 mm with a length of 50 mm with the corresponding length of the platform with a polymer substrate. *T_ext_* = 493 K, $v_{y}$ = 40 mm/s. Deposition was performed in both directions. The initial and boundary conditions are similar to those shown in Figure 2. The power of the heat source in accordance with Table 3 is accepted $P_{0}$ = 1.05 W. As a result of solving the thermal problem, it was found that the temperature *T_w_* = 475 K in the contact zone between the 9th and 10th layer exceeds the melting temperature *T_l_* of PLA, which indicates an excessive amount of heat in the system. It can lead to thermal deformations of the sample. Figure 7b shows the results of a numerical study of the thermal field, where the overheating of the sample layers is visualized. A graph of the evolution of temperature values at the point (0.2, 25, 1.36) is shown in Figure 7c,d shows a graph of the evolution of the volume of the melt pool, on which you can see spikes during the return movement of the heat source and the overall increase in the volume of the pool.

Verification of this stage of modeling was performed as a part of the experiment on deposition of short samples with a width of one bead from PLA without temperature regulation.

Short samples (Figure 7) were printed with a length of 50 mm and a height of 15 mm (100 layers). The deposition of the samples was performed both in one direction and in both directions (markings on the surface of the samples O—in one direction, and TO—in both directions). The samples were printed at two different printing speeds *v* = 10 mm/s (marking st4 or IV) and 40 mm/s (marking st2 or II). 

**Figure 8:**
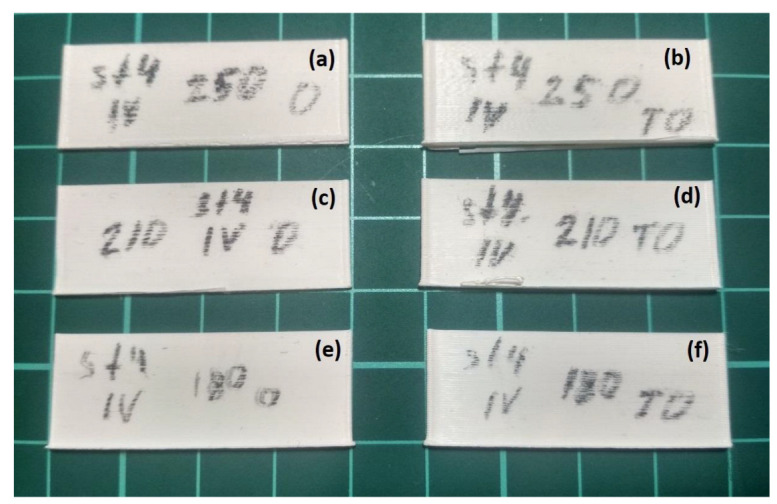
The external appearance of short samples printed at a printing speed of *v* = 10 mm/s: (**a**) sample printed in one direction at *T_ext_* = 523 K; (**b**) sample printed in both directions *T_ext_* = 523 K; (**c**) sample printed in one direction at *T_ext_* = 483 K; (**d**) sample printed in both directions *T_ext_* = 483 K; (**e**) sample printed in one direction at *T_ext_* = 453 K; (**f**) sample printed in both directions *T_ext_* = 453 K.

**Figure 9:**
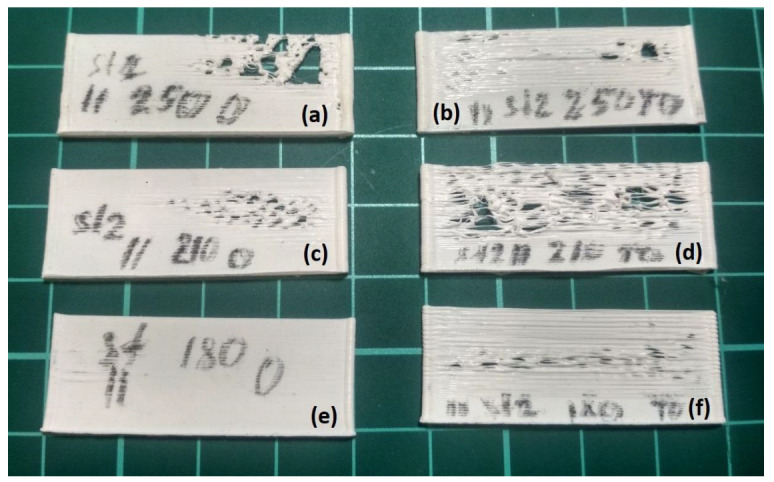
The external appearance of short samples printed at a printing speed of *v* = 40 mm/s: (**a**) sample printed in one direction at *T_ext_* = 523 K; (**b**) sample printed in both directions *T_ext_* = 523 K; (**c**) sample printed in one direction at *T_ext_* = 483 K; (**d**) sample printed in both directions *T_ext_* = 483 K; (**e**) sample printed in one direction at *T_ext_* = 453 K; (**f**) sample printed in both directions *T_ext_* = 453 K.

Figure 8 shows the external appearance of short samples printed at a printing speed of *v* = 10 mm/s (st4 or IV). The samples printed in one direction (O) are grouped in the left column, and the samples printed in both directions (TO) are grouped in the right column. In the lower row there are samples printed at the extrusion temperature *T_ext_* = 453 K, in the middle row at *T_ext_* = 483 K, in the upper row at *T_ext_* = 523 K. Figure 9 shows the external appearance of short samples printed at a printing speed of *v* = 40 mm/s (st2 or II), which are grouped in a similar way.

The external appearance of the short samples shown in Figure 8 demonstrates the absence of pronounced thermal deformations, which indicates sufficient cooling time of the previous layers at a printing speed of *v* = 10 mm/s for any temperature values *T_ext_*. At the same time, among the samples presented in Figure 9, thermal deformations are absent only in the sample, which was printed in one direction at the lowest permissible temperature *T_ext_* = 453 K. For other samples, which were printed in one direction, thermal deformations are typical, and they concentrate at the end of the layer, as predicted by the results of numerical simulation. In addition, the samples printed in both directions are characterized by pronounced thermal deformations along the entire length of the layer starting from a height of approximately 3 mm. The results of this experiment are fully consistent with the results of modeling the process of deposition a short sample at *T_ext_* = 493 K and $v_{y}$ = 40 mm/s, shown in Figure 7, where the welding temperature *T_w_* exceeded the melting point *T_l_* of PLA starting from the 10th layer. Thus, samples with a length of 50 mm are characterized by overheating of the polymer material. As a result, the previous layers do not have time to harden sufficiently and break off from each other by a new applied layer of material, forming lumps and blobs, thereby destroying the structure of the sample mainly closer to the center, where the sample overheats the most. 

### 3.6. Modeling of the FFF Printing Process of a Long Sample according to the Computational Scheme 2 for T_ext_ = 453 K 

The next step was to model the deposition of the long sample completely similar to the shown in Figure 2 with identical boundary and initial conditions. During deposition, the minimum extrusion temperature at which the layers are welded is accepted: *T_ext_* = 453 K. The deposition was performed in one direction at $v_{y}$ = 40 mm/s. The power of the heat source in accordance with Table 3 is accepted as $P_{0}$ = 0.85 W.

**Figure 10:**
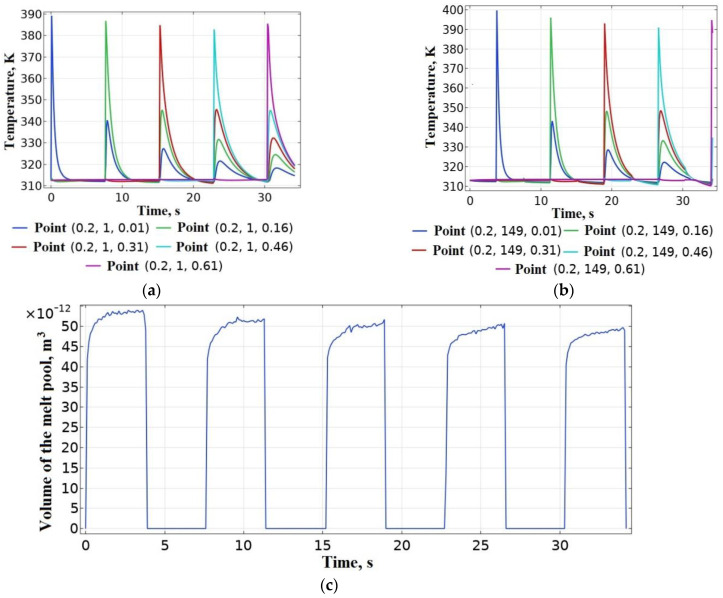
Modeling of long sample deposition: (**a**) evolution of temperature values in the contact zones at the beginning of the layer; (**b**) evolution of temperature values in the contact zones at the end of the layer; (**c**) evolution of the volume of the melt pool.

As a result of solving the thermal problem, the evolution of temperature values in the contact zones between layers at the beginning and end of the layer is studied. The results shown in Figure 10a demonstrate that the welding temperature *T_w_* at the beginning of the layer fluctuates around the crystallization temperature *T_c_* = 378 K of PLA, which indicates the possible absence of a weld between the previous and subsequent layer. At the same time, Figure 10b shows that at the end of the layer *T_w_* > *T_c_*, which indicates the possibility of creating a weld and the accumulation of heat at the end of the sample. Figure 10c shows a graph of the evolution of the volume of the melt pool, which also reports insufficient heating at the beginning of the previous layer and gradual overheating of the end of the layer. At the same time, there is a gradual decrease in the volume of the melt pool, which reaches a steady state by the 5th layer.

The current stage of modeling has shown the need to regulate the power of the heat source in the FFF printing process, due to insufficient heating or overheating of the material layers, using the simplest typical elements: rectangular samples with a width of one bead, on which these effects manifest themselves most clearly. To confirm these conclusions, further verification of the models will be performed on the basis of our own experiment.

### 3.7. Modeling of the FFF Printing Process of a Long Sample Using a PI Controller in the Algorithm of Numerical Implementation 

The last stage is a numerical simulation of the process of deposition the sample according to the computational scheme shown in Figure 2, but with the inclusion of real-time control in the algorithm of numerical implementation, namely the PI control law, which was formulated by the using of Equation (5). 

**Figure 11:**
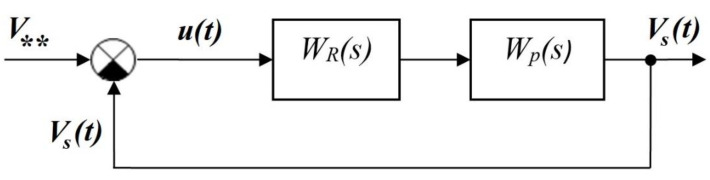
Block diagram of a closed-loop ACS of the melt pool volume.

To develop an automatic control system (ACS) for the power of a heat source in the process of deposition with feedback on the volume of the melt pool, it is necessary to determine the characteristics of the plant, the coefficients of the PI controller, and determine the stability of the system. During the development of the ACS, the melt pool itself acts as the plant. Melt pool volume $V_{**}$, which will be used in a closed-loop ACS as the setpoint (input), was determined as a result of modeling the deposition of the first layer of thermoplastics on a platform with a polymer substrate according to the computational scheme shown in Figure 2. The shape of the curve of the evolution of the melt pool volume (Figure 4a) in an open-loop ACS with a step input on the plant could be treated to a first order plant [111,112]. The gain *k*_0_ = 6.76 × 10^−11^ of the plant was determined as the ratio of the steady-state process value (output) $V_{**}$ to the stimulus (input) $P_{0}$ in an open-loop ACS. The setpoint value (input) in an open-loop ACS was the power of the heat source $P_{0}$ = 1.05 W, corresponding to the heating of the bead from PLA to *T_ext_* = 493 K. The process value (output) of the volume of the melt pool was $V_{**}$ = 7.1× 10^−11^ m^3^ (Figure 4a). The value of the time constant *T*_1_ according to the plant response to a step input was 0.025 s (Figure 4a). The closed-loop ACS of the volume of the melt pool was considered as linear. A classic PI controller was used. The block diagram of a closed-loop ACS is shown in Figure 11.

In a closed-loop ACS, the specified value of the volume of the melt pool is taken as a setpoint (input) $V_{**}$, and the actual value of the volume of the melt pool is taken as a process (output) value $V_{s}(t)$. The transfer function of the PI controller *W_R_(s)* is described by Equation (6).

(6) $$W_{R}\left(s\right)=K_{p}+K_{i}/s,$$

*u(t)* is the magnitude error between the setpoint value of the pool volume $V_{**}$ and a feedback signal (measured pool volume $V_{s}(t)$).

The transfer function of the plant *W_P_(s)* is described by Equation (7).

(7) $$W_{p}(s)=k_{0}/\left(T_{1}s+1\right).$$

Modeling the operation of a closed-loop ACS in the Simulink application of the Matlab package made it possible to determine the coefficients of the PI controller, taking into account the discrete nature of the process. The discrete time for PI controller was assumed as follows τ = 0.01 s. The coefficients of the PI controller after calculation were $K_{p}=0.017$, $K_{I}=0.635$ c^−1^ and were used to implement real-time control at the last stage of modeling in the Comsol Multiphysics package.

**Figure 12:**
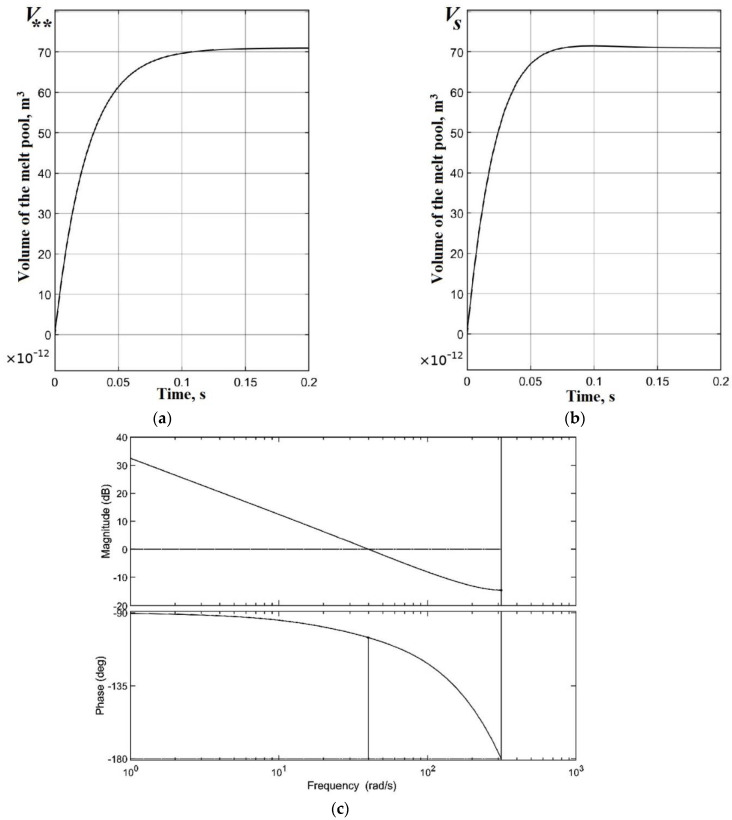
Results of the modeling of the ACS of the melt pool volume in the Simulink application: (**a**) transient response in an open-loop ACS; (**b**) transient response in a closed-loop ACS; (**c**) the open-loop Bode magnitude and phase plots.

Figure 12 shows the simulation results in the Simulink application of the Matlab package. Figure 12a,b show estimated transients in open-loop and closed-loop ACS. The settling time in a closed-loop ACS is 0.06 s, and the maximum overshoot does not exceed 3%, the steady-state error is 0. The open-loop Bode magnitude and phase plot of the melt pool volume demonstrates the stability of the closed-loop ACS (Figure 12b). The phase margin is 74.7°, and the gain margin is 14.7 dB.

**Figure 13:**
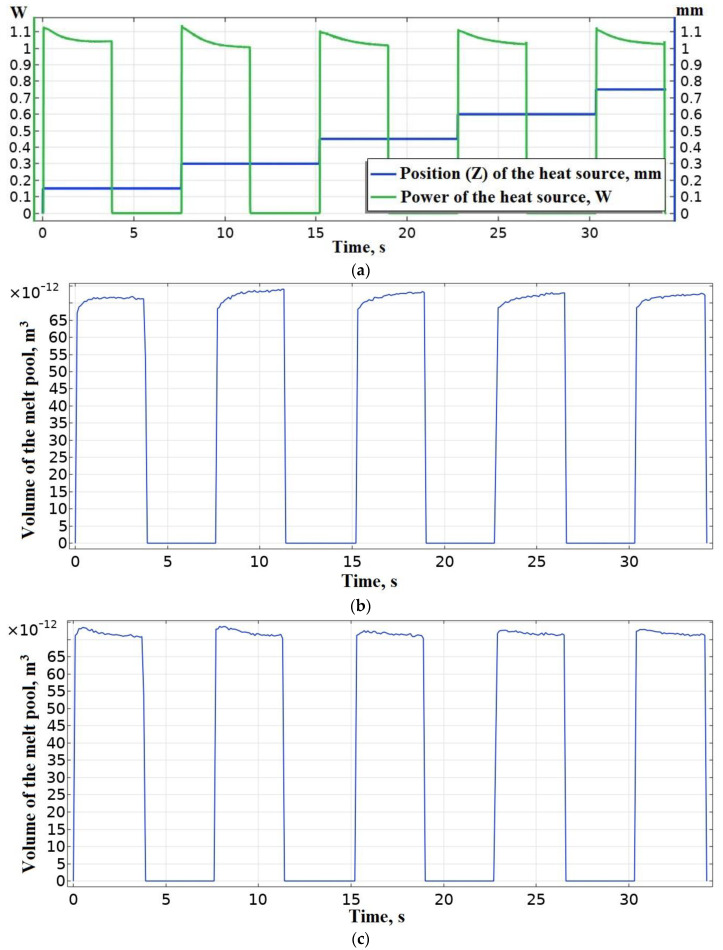
The result of solving a thermal problem with real-time control of the power of the heat source: (**a**) the desired function of the power of the heat source from time; (**b**) a graph of the evolution of the volume of the melt pool in an open-loop ACS at *T_ext_* = 493 K; (**c**) a graph of the evolution of the volume of the melt pool in a closed-loop ACS.

The results of the modeling stage of the sample deposition process according to the computational scheme shown in Figure 2, with the real-time control (PI controller) of the heat source power included in the algorithm of numerical implementation (Equations (1)–(5)) are presented in Figure 13.

As a result of solving the thermal problem, a function of the power of the heat source from time was obtained (Figure 13a). It was then compared with data on the coordinates and speed/time of movements of the extruder and, thus, used to regulate the extrusion temperature during the deposition process. Before using it in a real deposition cycle, the resulting function can be smoothed using software tools.

The results presented in Figure 13c demonstrate a stable process of sample formation with a sufficiently stable volume of the melt pool. Such control of the heat source power enables preventing overheating and insufficient heating of the previous layers and, thus, ensures a high and stable quality of welds both inside the layer and between layers.

The graph shown in Figure 13a demonstrates the requirement for a gradual decrease in the extrusion temperature as the sample layer is made. At the same time, a higher extrusion temperature is required for deposition the first layer, which is due to the heat removing properties of the glass platform. The similar influence of the platform decreases with each layer. The thermal input of the platform was considered in detail during the analysis and minimized in this study. After the system enters the steady state on the fifth layer, the required power reduction inside one layer is approximately 11% (from 1.13 to 1.01 W) with a layer deposition duration of 3.8 s. At the same time, it is known from [101] that the cooling time of a lightweight nozzle (<1 g) on 20 degrees is 3 s in the temperature range from 473 to 573 K without extrusion, and will be 10% when cooled from 493 to 473 K, which meets the requirements presented.

Verification of the modeling stages for the deposition of long samples (Figure 4, Figure 10 and Figure 13) was performed as a part of the experiment on the deposition of long samples with a width of one bead from PLA without temperature regulation and with temperature regulation during deposition in accordance with the obtained settings (Figure 13a).

**Figure 14:**
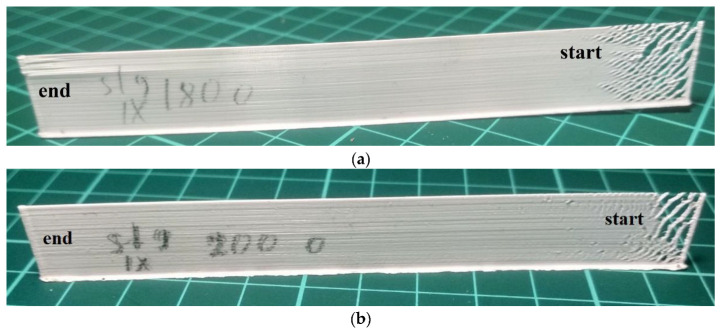
External appearance of long samples printed in one direction (*v* = 40 mm/s): (**a**) sample printed at *T_ext_* = 453 K; (**b**) sample printed at *T_ext_* = 473 K.

Long samples were printed (Figure 2) with a length of 150 mm and a height of 20 mm (133 layers) without the regulation of the extrusion temperature *T_ext_*. The samples were printed in one direction at a printing speed of *v* = 40 mm/s. The external appearance of the long sample shown in Figure 14a demonstrates an accumulating defect associated with insufficient temperature *T_ext_*. It is fully consistent with the modeling results shown in Figure 8. In Figure 8, at *T_ext_* = 453 K at the beginning of the layer, the welding temperature *T_w_* fluctuated according to the crystallization temperature *T_c_* with PLA and did not guarantee the creation of a weld with the previous layer, and the graph of the evolution of the melt pool (Figure 8b) showed its small volume and slow growth from the beginning to the end of the layer. The same effect can be observed in Figure 13b during the modeling the sample printing process for *T_ext_* = 493 K. It is important to note that a long sample printed at *T_ext_* = 473 K (Figure 14b) is also characterized by a defect associated with an insufficient value of *T_ext_*, but expressed to a lesser extent.

**Figure 15:**
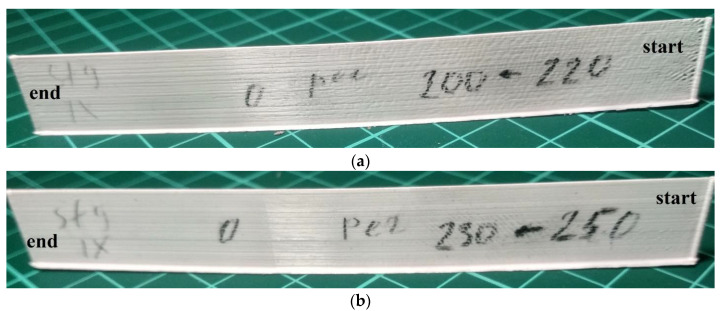
External appearance of long samples printed in one direction (*v* = 40 mm/s) with the regulation of the extrusion temperature: (**a**) regulation of *T_ext_* from 493 to 473 K; (**b**) regulation of *T_ext_* from 523 to 503 K.

Next, long samples were printed (Figure 2) with a length of 150 mm and a height of 20 mm (133 layers) with the regulation of the extrusion temperature *T_ext_*. The samples were printed in one direction at a printing speed of *v* = 40 mm/s. The heat source power function obtained as a result of modeling (Figure 13a) was used to print samples in two versions: with regulation from 493 to 473 K and from 523 to 503 K (Figure 15a,b).

The external appearance of the long sample shown in Figure 15a also demonstrates the presence of a defect associated with insufficient *T_ext_* at the beginning of the layer, but to a much lesser extent. The deposition of this sample was performed with the regulation of the extrusion temperature from 493 K at the beginning of the layer to 473 K by the end of the layer. In addition, it is possible to note the unevenness of the surface, which is also typical of the samples presented in Figure 14.

The external appearance of the long sample shown in Figure 15b demonstrates the complete absence of a defect associated with an insufficient value of *T_ext_*, as well as a flat surface. The deposition of this sample was performed with the regulation of the extrusion temperature from 523 K (close to the destruction temperature of PLA) at the beginning of the layer up to 503 K by the end of the layer. In this case, a gradual decrease in the temperature *T_ext_* from the beginning to the end of the layer also made it possible to avoid overheating of the material at the end of the layer and possible defects caused by long-term continuous operation at temperatures close to the destruction temperature of PLA. Thus, the results of the experiment showed the effectiveness of the proposed approach for regulating the extrusion temperature to ensure stable and high quality of welds between the beads of thermoplastic material on the example of PLA in the FFF printing process. The adequacy of the developed mathematical model is shown and the possibility of its use for the layer-by-layer manufacturing of products is confirmed. 

## 4. Conclusions

The mathematical model of the FFF printing process has been developed with the use of real-time control in the algorithm of numerical implementation. The successful solution of the thermal conductivity problem made it possible to determine segment-wise heating settings for use during the printing process, resulting in a high and stable quality of welding.

Verification of the model by comparison with other well-known mathematical models of the additive manufacturing process (FFF/FGF) by extrusion of thermoplastic material showed a deviation of approximately 12–15%. Verification of the model using the data of a deposition experiment of a double high wall showed a deviation of the results of the numerical simulation from the results of thermography by 17.7% during the measurement of the welding temperature between the last deposited layers. This indicates the adequacy of the proposed model.

The proposed model was verified with our own experiment using rectangular 3D polylactide shapes (samples) printed with and without regulation of the power of the heat source according to the previously estimated settings. Such a sample is a wall with a width of one bead and other specified dimensions. The results of the experiment fully correspond with the modeling results. The high quality of the samples printed using the developed method of controlling the thermal cycle of the process is shown. The results of an experiment demonstrated the regularities in the appearance of defects caused by insufficient heating or overheating of the polymer material during the FFF printing process.

A closed-loop ACS of the FFF printing process with the feedback on the volume of the melt pool has been developed. A simulation model of a closed-loop ACS was created. This made it possible to determine the PI coefficients of the controller and ensure high quality regulation. The stability of a closed-loop ACS is shown.

The results of solving the thermal conductivity problem can be used to analyze the thermal cycle of the FFF printing process as a three-dimensional problem. They can be applied to study the degree of coalescence and healing in the entire volume of the product using the well-known mathematical models of Frenkel, Eshelby, Pokluda, Defauchi, and De Gennes.

The development of thermal cycle control tools for the FFF printing process will make it possible to reduce thermal deformations and stresses, as well as improve the mechanical properties of products. 

In future research, we will consider using a different approach to FEM modeling such as voxel-based FEM algorithms, which enable us to proceed with the activation (“killing” and “aliving”) of separate elements (parts of the bead) instead of the whole layer at once.

## Figures and Tables

**Table 1:** Thermophysical constants of PLA accepted in the mode.

Constant	Units	Value
Specific heat capacity	J·kg^−1^·K^−1^	1800 [7,72,104]
Mass density	kg·m^−3^	1240 [72,92]
Coefficient of thermal conductivity	W·m^−1^·K^−1^	0.2 [67,92,104]
Heat transfer coefficient	W·m^−2^·K^−1^	20 [92,105]
Coefficient of thermal radiation	-	0.78 [57,67]

**Table 2:** Thermophysical constants of glass accepted in the model.

Constant	Units	Value
Specific heat capacity	J·kg^−1^·K^−1^	730
Mass density	kg·m^−3^	2210
Coefficient of thermal conductivity	W·m^−1^·K^−1^	1.4

**Table 3:** Results of solving the thermal problem for a single bead of material with different deposition parameters.

Extrusion Temperature *T_ext_*, K	$\mathrm{Deposition}\;\mathrm{Speed}\;v_{y}$, mm/s	Layer Height *l_z_*, mm	Layer (Bead) Width *l_x_*, mm	$\mathrm{Heat}\;\mathrm{Source}\;\mathrm{Power}\;P_{0}$, W
453	40	0.15	0.4	0.85
493	40			1.05
493	10			0.27
463	10	0.3	0.5	0.56

## Data Availability

The data presented in this study are available on request from the corresponding author. The data are not publicly available due to the fact that further study will be carried out using the same data.
